# Associations between mental health and lifestyle changes during the COVID-19 pandemic in a general Japanese population: NIPPON DATA2010

**DOI:** 10.1265/ehpm.24-00292

**Published:** 2025-04-25

**Authors:** Naoki Aono, Aya Higashiyama, Harumitsu Suzuki, Akira Fujiyoshi, Makiko Abe, Atsushi Satoh, Hisatomi Arima, Nobuo Nishi, Aya Kadota, Takayoshi Ohkubo, Tomonori Okamura, Nagako Okuda, Akira Okayama, Katsuyuki Miura

**Affiliations:** 1 Department of Hygiene, Wakayama Medical University, Wakayama, Japan; 2 Department of Preventive Medicine and Public Health, Faculty of Medicine, Fukuoka University, Fukuoka, Japan; 3 The Laboratory of Epidemiology and Prevention, Kobe Pharmaceutical University, Kobe, Japan; 4 Graduate School of Public Health, St. Luke’s International University, Tokyo, Japan; 5 NCD Epidemiology Research Center, Shiga University of Medical Science, Shiga, Japan; 6 Department of Public Health, Shiga University of Medical Science, Shiga, Japan; 7 Department of Hygiene and Public Health, Teikyo University School of Medicine, Tokyo, Japan; 8 Department of Preventive Medicine and Public Health, Keio University School of Medicine, Tokyo, Japan; 9 Division of Applied Life Sciences, Graduate School of Life and Environmental Sciences, Kyoto Prefectural University, Kyoto, Japan; 10 The Research Institute of Strategy for Prevention, Tokyo, Japan

**Keywords:** COVID-19, Pandemic, Psychological distress, Kessler 6, Lifestyle changes, Alcohol, Physical activity, NCDs, Japan, Nation

## Abstract

**Background:**

Deterioration in lifestyle associated with poor mental health could be an important concern during the coronavirus disease 2019 (COVID-19) pandemic. However, few studies have investigated the association between mental health status and lifestyle changes during the pandemic in nationwide Japanese general population.

**Methods:**

This cross-sectional study was conducted using the data among 1,546 participants of the follow-up study of NIPPON DATA2010 in 2021. Recent mental status, as assessed using the Kessler 6 (K6) scale, and lifestyle changes compared to before the pandemic were determined using self-reported questionnaires. Some lifestyle changes such as decreased physical activity were defined as undesirable, whereas others such as decreased alcohol drinking were defined as desirable. The participants were divided into three groups based on the K6 scores: the K6<5, 5≤K6<9, and K6≥9 groups. The odds ratios (ORs) and 95% confidence intervals (CIs) of the K6 groups for each lifestyle change compared with that in the K6<5 group were estimated after adjusting for possible confounders.

**Results:**

The ORs of the K6≥9 group for all undesirable lifestyle changes were significantly high, especially increased alcohol drinking (OR 4.64; 95% CI, 2.71–7.93), and decreased physical activity (OR 4.63; 95% CI, 3.29–6.52). Among the desirable changes, the OR of the 5≤K6<9 group was significantly high for increased eating home cooking.

**Conclusions:**

Poor mental health showed a significant association with undesirable lifestyle changes, especially increased alcohol drinking and decreased physical activity, in a nationwide general Japanese population during the COVID-19 pandemic.

**Supplementary information:**

The online version contains supplementary material available at https://doi.org/10.1265/ehpm.24-00292.

## Graphical Abstract

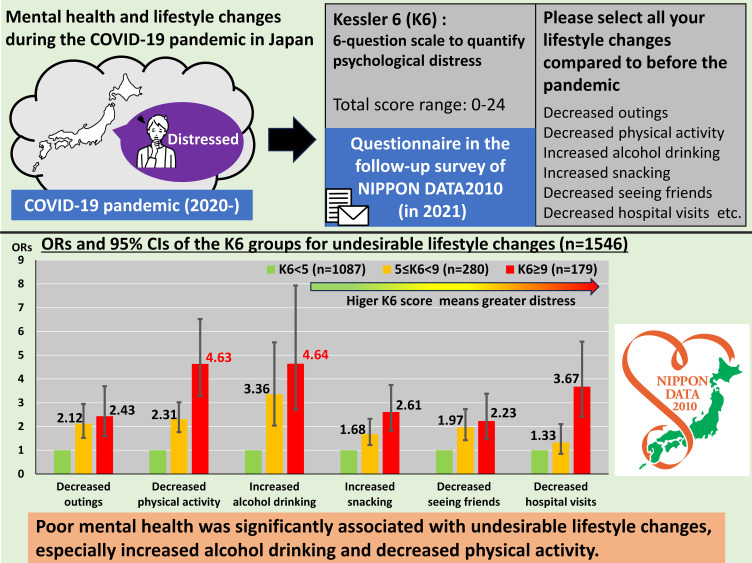

## Introduction

The spread of coronavirus disease 2019 (COVID-19), which subsequently became a pandemic, led to the World Health Organization declaring a Public Health Emergency of International Concern in 2020 [1]. Restrictive measures were adopted by the governments of several countries to prevent the spread of the pandemic, and these measures limited social activities and worsened the mental health status of individuals in countries that implemented lockdowns [2, 3]. Moreover, similar effects were observed in countries such as Japan, where restricted internal movement was considered a more moderate measure than lockdown [4].

Implementing measures for the prevention of infection is a priority during pandemics. However, maintaining a good lifestyle is important for prevention of non-communicable diseases (NCDs). Poor mental health has been associated with undesirable lifestyle changes, such as increased alcohol drinking [5] and overeating [6], even before the COVID-19 pandemic. Thus, the deterioration of lifestyle associated with poor mental health could be an important concern during the COVID-19 pandemic.

In previous studies among individuals including Japanese workers, poor mental health was associated with undesirable lifestyle changes during the COVID-19 pandemic, such as increased alcohol drinking [7, 8] and decreased physical activity [8, 9]. However, few studies have investigated these associations among nationwide general population in Japan, wherein the incidence of COVID-19 infection was relatively lower [10] and behavioral restrictions were less severe than those in Western countries [11, 12].

This cross-sectional study in the participants of the National Integrated Project for Prospective Observation of Non-communicable Disease and its Trends in the Aged 2010 (NIPPON DATA2010) aimed to investigate the association between mental health status and lifestyle changes during the COVID-19 pandemic in the general population in Japan [13].

## Methods

### Participants

The study population comprised the participants of the NIPPON DATA2010, a prospective cohort study of cardiovascular diseases. A total of 8,815 residents aged 1 year or older from 300 randomly selected districts across Japan participated in the National Health and Nutrition Survey of Japan (NHNS2010) in November 2010. Among the 7,229 participants of NHNS2010 aged ≥20 years, 3,873 (1,598 men and 2,275 women) underwent a blood test, and 2,898 (1,239 men and 1,659 women) participated in the baseline NIPPON DATA2010 survey [13, 14].

**Fig. 1:**
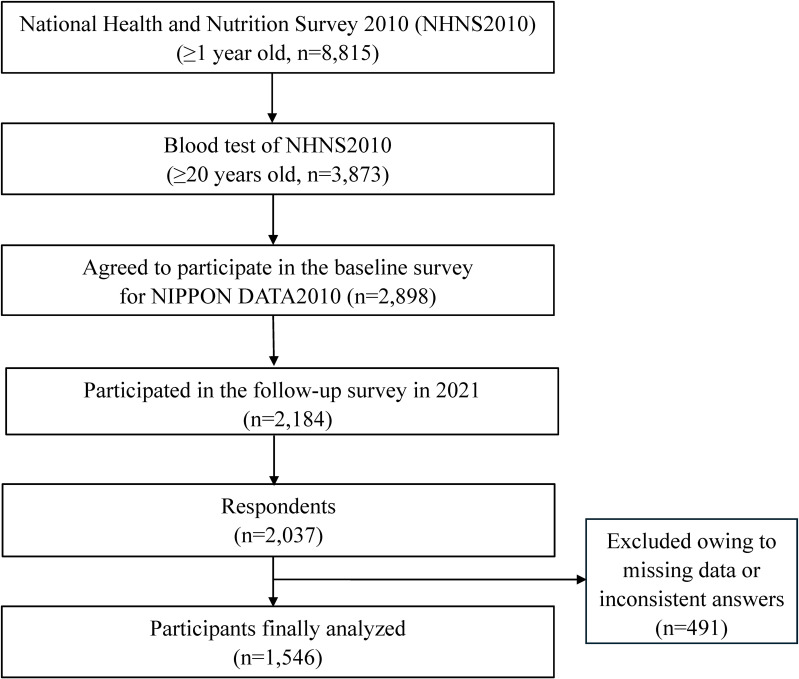
Flowchart of participant recruitment

The follow-up survey of the NIPPON DATA2010 annually collected information on the incidence of stroke, heart disease, and diabetes mellitus, and medication for hypertension and dyslipidemia, using a self-administered questionnaire via mail or telephone interviews [13]. In the follow-up survey in autumn 2021, 2,184 participants were also asked about their current mental health status using the Kessler 6 (K6) scale, and lifestyle changes compared to before the COVID-19 pandemic. Among the 2,037 participants who responded to the survey, 491 were excluded from the present study owing to missing data or inconsistent answers. Thus, the final analysis included 1,546 participants (Fig. 1).

### Data collection

Data regarding sex, age, and education (low: no education/elementary school/junior high school level; middle: high school level; and high: junior college/university level) [15, 16] were collected from the baseline survey of NIPPON DATA2010.

The Japanese version of the K6 scale, which is used to quantify non-specific psychological distress [17, 18], was included in the self-administered questionnaire of the follow-up survey in 2021. K6 comprises the following six questions: during the past 30 days, how often did the respondent feel: 1) nervous, 2) hopeless, 3) restless or fidgety, 4) so depressed that nothing could cheer them up, 5) that everything was an effort, and 6) worthless. The responses to these questions were scored as follows: “0: None of the time”, “1: A little of the time”, “2: Some of the time”, “3: Most of the time” and “4: All of the time”, with a total score ranged 0–24 [17, 19, 20]. A higher score indicates greater distress. The participants were divided into three groups based on the K6 score: the K6<5, 5≤K6<9, and K6≥9 groups. The cut-off score for K6 was set as 5 to identify psychological distress [21, 22]. A score of 9 indicates probable mood/anxiety disorder [18, 22].

Lifestyle changes compared to before the COVID-19 pandemic were evaluated using 10 questionnaires of the follow-up survey in 2021. The participants selected all of the followings that applied to them: decreased frequency of going out (decreased outings), decreased frequency of physical activity (decreased physical activity), increased frequency or amount of alcohol consumption (increased alcohol drinking), decreased frequency or amount of alcohol consumption (decreased alcohol drinking), increased frequency or amount of vegetable intake (increased vegetable intake), increased frequency or amount of snacking (increased snacking), increased frequency of eating home cooking (increased eating home cooking), decreased frequency of seeing friends in person (decreased seeing friends), and coming to refrain from visiting hospital (decreased hospital visits).

The cumulative number of cases of COVID-19 per 100,000 individuals between 15 Jan 2020 (the start date of reporting cases) and 31 Oct 2021 in 47 prefectures in Japan was calculated using the data obtained from the Ministry of Health, Labour and Welfare (MHLW) [23]. The prefectures were divided into quartile groups according to the incidence of COVID-19, and the participants were classified into quartiles based on their addresses [24]. Six prefectures, including Tokyo, were classified into the highest infected area (Q4, 1,569–3,425 cases/100.000, n = 412). Nine prefectures were classified into Q3 (877–1,451 cases/100,000 n = 390). Fourteen prefectures were classified into Q2 (583–846 cases/100,000, n = 374). Eighteen prefectures were classified into Q1 (196–578 cases/100,000, n = 370).

### Statistical analysis

The characteristics of the participants of the follow-up survey in 2021 were compared among the K6 groups. The age of the participants in 2021 was obtained by adding 11 to age at the time of the baseline survey. One-way analysis of variance was used to analyze age. Categorical variables were assessed using chi-squared tests. To determine the association between mental health status and lifestyle changes compared with that before the pandemic, the odds ratios (ORs) and their 95% confidence intervals (CIs) of the K6 groups for each lifestyle change compared with those of the K6<5 group were estimated using age-sex adjusted and multivariable-adjusted logistic regression model after adjusting for the following potential confounding factors: age, sex, education, quartiles of prefectures by the COVID-19 cumulative incidence, medication for hypertension and/or dyslipidemia within a year, and indication of diabetes or hyperglycemia within a year. The lifestyle changes were divided into two groups: undesirable and desirable. The undesirable lifestyle changes included decreased outings, decreased physical activity, increased alcohol drinking, increased snacking, decreased seeing friends, and decreased hospital visits. The desirable lifestyle changes included decreased alcohol drinking, increased vegetable intake, and increased eating home cooking [25–28]. In addition, we conducted sex-specific analyses and age-stratified analyses in older participants (aged ≥65 years) and younger participants (aged <65 years).

All P-values were two-sided. A P-value of <0.05 was considered statistically significant. All statistical analyses were performed using SAS software (version 9.4; SAS Institute Inc., Cary, NC, USA).

## Results

The mean age of the participants was 65.9 (standard deviation [SD], 14.5) years, and 40.3% of the participants were men (623 men and 923 women). The proportion of the participants in each K6 group was as follows: K6<5, 70.3%; 5≤K6<9, 18.1%; and K6≥9, 11.6%.

Table 1 presents the characteristics of the participants according to the K6 groups. The mean age was lower in the groups with higher K6 scores. The quartiles of prefectures by the COVID-19 cumulative incidence showed no association with the K6 groups. The proportion of the participants starting medication for dyslipidemia within a year was the highest in the 5≤K6<9 group (26.8%).

**Table 1:** Characteristics of the study participants according to the K6 group (n = 1546)

	**K6 group**			**P-values^a^**

	**K6<5** **(n = 1087)**	**5≤K6<9** **(n = 280)**	**K6≥9** **(n = 179)**	
Sex, n (%)				
Men	456 (42.0)	108 (38.6)	59 (33.0)	0.061
Women	631 (58.1)	172 (61.4)	120 (67.0)	
Age, years, mean (SD)	66.4 (14.1)	65.8 (14.7)	63.1 (16.2)	0.019
Education, n (%)				
Low	165 (15.2)	44 (15.7)	30 (16.8)	0.614
Middle	507 (46.6)	125 (44.6)	72 (40.2)	
High	415 (38.2)	111 (39.6)	77 (43.0)	
Quartiles of prefectures by the COVID-19 cumulative incidence, n (%)				
Quartile 1 (lowest: 18 prefectures)	265 (24.4)	63 (22.5)	42 (23.5)	0.786
Quartile 2 (14 prefectures)	266 (24.5)	68 (24.3)	40 (22.4)	
Quartile 3 (9 prefectures)	261 (24.0)	79 (28.2)	50 (27.9)	
Quartile 4 (highest: 6 prefectures)	295 (27.1)	70 (25.0)	47 (26.3)	
Medication for hypertension^b^, n (%)	360 (33.1)	98 (35.0)	56 (31.3)	0.703
Medication for dyslipidemia^b^, n (%)	219 (20.2)	75 (26.8)	35 (19.6)	0.045
Diabetes or hyperglycemia^b^, n (%)	96 (8.8)	31 (11.1)	20 (11.2)	0.377

K6, Kessler 6; SD, standard deviation.

^a^Chi-squared tests for categorical variables and analysis of variance for age.

^b^Self-reported

Table 2 presents the proportion of participants with each undesirable lifestyle change by K6 group, and age-sex adjusted and the multivariable-adjusted ORs of the K6 groups for each undesirable lifestyle change. Approximately 80% of the participants in the 5≤K6<9 and K6≥9 groups reported experiencing undesirable lifestyle changes such as “decreased outings” and “decreased seeing friends”. The odds of reporting undesirable lifestyle changes were higher in a dose-response manner in the worse K6 groups. The strength of the associations with worse K6 groups was particularly high for the following: increased alcohol drinking (OR 3.36; 95% CI, 2.04–5.54 in the 5≤K6<9 group, and OR 4.64; 95% CI, 2.71–7.93 in the K6≥9 group) and decreased physical activity (OR 2.31; 95%CI, 1.76–3.02 in the 5≤K6<9 group and OR 4.63; 95% CI, 3.29–6.52 in the K6≥9 group). The sex-specific analyses yielded similar results (Supplementary Table 1). Age-stratified analyses revealed that the ORs of older participants were higher than those of younger participants for almost all undesirable lifestyle changes (Supplementary Table 2).

**Table 2:** Odds ratios and 95% confidence intervals of the K6 groups for undesirable lifestyle changes (n = 1546)

**Lifestyle changes**	**n**	**Lifestyle change (+)** **n (%)**	**Age-sex adjusted** **OR (95% CI)**	**Multivariable-adjusted** **OR (95% CI)^a^**
Decreased outings				
K6<5	1087	729 (67.1)	1.00	1.00
5≤K6<9	280	228 (81.4)	2.14 (1.54–2.97)	2.12 (1.52–2.95)
K6≥9	179	149 (83.2)	2.35 (1.55–3.56)	2.43 (1.60–3.69)
Decreased physical activity				
K6<5	1087	346 (31.8)	1.00	1.00
5≤K6<9	280	145 (51.8)	2.31 (1.77–3.02)	2.31 (1.76–3.02)
K6≥9	179	121 (67.6)	4.59 (3.26–6.45)	4.63 (3.29–6.52)
Increased alcohol drinking				
K6<5	1087	42 (3.9)	1.00	1.00
5≤K6<9	280	31 (11.1)	3.31 (2.02–5.43)	3.36 (2.04–5.54)
K6≥9	179	27 (15.1)	4.62 (2.71–7.87)	4.64 (2.71–7.93)
Increased snacking				
K6<5	1087	174 (16.0)	1.00	1.00
5≤K6<9	280	69 (24.6)	1.70 (1.24–2.34)	1.68 (1.22–2.32)
K6≥9	179	59 (33.0)	2.52 (1.77–3.59)	2.61 (1.82–3.74)
Decreased seeing friends				
K6<5	1087	731 (67.3)	1.00	1.00
5≤K6<9	280	225 (80.4)	1.99 (1.44–2.75)	1.97 (1.42–2.73)
K6≥9	179	148 (82.7)	2.15 (1.42–3.25)	2.23 (1.47–3.38)
Decreased hospital visits				
K6<5	1087	85 (7.8)	1.00	1.00
5≤K6<9	280	28 (10.0)	1.29 (0.82–2.02)	1.33 (0.85–2.10)
K6≥9	179	43 (24.0)	3.53 (2.34–5.33)	3.67 (2.42–5.57)

K6, Kessler 6; OR, odds ratio; CI, confidence interval.

^a^Adjusted for age, sex, education, quartiles of prefectures by the COVID-19 cumulative incidence, medication for hypertension and/or dyslipidemia within a year, and indication of diabetes or hyperglycemia within a year.

Table 3 presents the proportion of participants with each desirable lifestyle change by K6 group, age-sex adjusted and the multivariable-adjusted ORs (95% CI) of the K6 groups for each desirable lifestyle change. The proportion of participants in each K6 group reporting was as follows: decreased alcohol drinking, 7–12%; increased vegetable intake, 14–18%; and increased eating home cook, 38–46%. Only the OR for increased eating home cooking was significant in the 5≤K6<9 group (OR 1.39; 95% CI, 1.06–1.81). Sex-specific analyses yielded no significant OR (Supplementary Table 3). Age-stratified analyses revealed that the OR for increased eating home cooking of older participants in the 5≤K6<9 group was significantly high (OR 1.58; 95% CI, 1.11–2.25). However, the OR for decreased alcohol drinking in younger participants was significantly low (OR 0.38; 95% CI, 0.15–0.99) (Supplementary Table 4).

**Table 3:** Odds ratios and 95% confidence intervals of the K6 groups for desirable lifestyle changes (n = 1546)

**Lifestyle changes**	**n**	**Lifestyle change (+)** **n (%)**	**Age-sex adjusted** **OR (95% CI)**	**Multivariable-adjusted** **OR (95% CI)^a^**
Decreased alcohol drinking				
K6<5	1087	102 (9.4)	1.00	1.00
5≤K6<9	280	22 (7.9)	0.85 (0.52–1.37)	0.85 (0.52–1.38)
K6≥9	179	22 (12.3)	1.44 (0.87–2.37)	1.40 (0.85–2.33)
Increased vegetable intake				
K6<5	1087	188 (17.3)	1.00	1.00
5≤K6<9	280	41 (14.6)	0.82 (0.57–1.19)	0.81 (0.56–1.18)
K6≥9	179	33 (18.4)	1.14 (0.76–1.73)	1.12 (0.74–1.70)
Increased eating home cooking				
K6<5	1087	420 (38.6)	1.00	1.00
5≤K6<9	280	131 (46.8)	1.39 (1.06–1.81)	1.39 (1.06–1.81)
K6≥9	179	77 (43.0)	1.18 (0.85–1.62)	1.18 (0.85–1.63)

K6, Kessler 6; OR, odds ratio; CI, confidence interval.

^a^Adjusted for age, sex, education, quartiles of prefectures by the COVID-19 cumulative incidence, medication for hypertension and/or dyslipidemia within a year, and indication of diabetes or hyperglycemia within a year.

After excluding participants who have received medication for hypertension and/or dyslipidemia within a year or indication of diabetes or hyperglycemia within a year, the results were similar. A subgroup analysis was conducted, dividing the subgroup of the participants aged <65 years into two groups by median age (51 years). The tendencies of the results were almost similar to those aged ≥65 years (data not shown).

## Discussion

This cross-sectional study showed that poor mental health, as measured using the K6 scale, was associated with all the undesirable lifestyle changes in a dose-response manner during the COVID-19 pandemic in Japanese nationwide general population. Among undesirable lifestyle changes, the ORs for “increased alcohol drinking” and “decreased physical activity” were particularly high in the 5≤K6<9 and K6≥9 groups: alcohol drinking (3.36 and 4.64, respectively) and physical activity (2.31 and 4.63, respectively). Among the desirable lifestyle changes, a significantly higher OR was observed only for “increased eating home cooking” in the 5≤K6<9 group compared with that in the K6<5 group.

The appropriateness of the multivariable logistic regression model (generalized linear model) used in the study was supported by a goodness-of-fit test. In general, the strength of the logistic regression model includes ease of interpretation because the odds ratio is obtained based on the linear assumption. A limitation of the model, however, is that the obtained index is an odds ratio, which is not necessarily a good approximate value to a risk ratio, especially when the number of outcomes is not small enough relative to the number of non-outcome.

The ORs for increased alcohol drinking were the highest among those with undesirable lifestyle changes, i.e., the 5≤K6<9 and K6≥9 groups in the present study. In previous studies, strong associations were observed between poor mental health and increased alcohol drinking during the pandemic [8, 29–31]. Konno et al. reported that the OR of feeling loneliness for increased alcohol consumption during the pandemic was significantly high in Japanese workers (OR 1.88; 95% CI, 1.65–2.15). They considered that negative coping behaviors to suppress loneliness were possibly related to increased alcohol consumption [7]. A cross-sectional study in the U.S. reported a positive correlation between mental stress related to the COVID-19 pandemic and drinking alcohol to cope with stress (r = 0.61; p < 0.001) [32]. Studies conducted before the pandemic suggested that mental stress affects the reward system of the brain and causes alcohol cravings [33], while prior alcohol dependence was reported to be associated with current depressive symptoms [34]. Thus, mental stress and alcohol drinking may have affected each other bi-directionally in the present study. Furthermore, Konno et al. also reported that this association was observed regardless of the frequency of alcohol drinking before the pandemic [7]. Alcohol abuse may have been associated with the prevalence of family violence during the pandemic [35, 36]. Thus, increased alcohol drinking associated with mental stress should be a public health priority during future pandemics, regardless of the usual frequency of alcohol drinking.

The ORs for decreased physical activity were the second highest among the ORs for undesirable lifestyle changes in the present study. Previous studies have investigated the associations between poor mental health and physical activities during the COVID-19 pandemic [8, 29, 30, 37]. A cross-sectional study in Czech reported that the OR of deterioration of mental health related to the pandemic for decreased physical activity was significantly high (OR 1.67; 95% CI, 1.21–2.28) [9]. A cross-sectional study among older adults in Japan also reported that poor mental health, as assessed using the 12-Item Short-Form Health Survey version 2, revealed significant associations with decreased physical activity during the pandemic. The authors considered that low health literacy among individuals with poor mental health prevented them from integrating information related to COVID-19 [38]. In addition, several previous studies have suggested that the decrease in physical activity during the pandemic may be attributed to the closure of exercise facilities [30, 38]. Consequently, the participants who were unable to maintain their physical activity level owing to the closure of the facilities may have experienced deterioration in their mental health status in the present study. Thus, in future pandemics, it will be both important for health care providers to select individuals with high risk for decreased physical activities such as those with poor mental health, and to help them in developing environment where they can exercise inside or outside the home and in promoting health literacy among them.

Poor mental health showed a significant association with increased snacking in the present study. Snacking was not clearly defined in the present study; however, ultra-processed food (UPF) is a snack in Japan. A cross-sectional study in Italy reported that psychological distress showed a significant association with increased UPF intake during the lockdown and negatively impacted diet quality [39]. COVID-19-related stress was also reported to be correlated with eating to cope with stress (r = 0.64; p < .001) and increased sugar intake (r = 0.28; p < .001) [32]. As with increased alcohol drinking, poor mental health may have increased the frequency of snacking, including the consumption of sugary and highly palatable processed foods, to cope with stress in the present study.

The association between poor mental health and increased vegetable intake as a desirable lifestyle change was not significant in the present study. However, previous studies have shown that poor mental health was associated with decreased vegetable intake during the pandemic [40, 41]. The answers to the items in the questionnaire did not include “decreased vegetable intake” as a response in the present study; therefore, the association between mental health status and vegetable intake could not be thoroughly investigated in this study.

The OR for “increased eating home cooking” in the 5≤K6<9 group was significantly high in the present study. The pandemic had a negative economic impact on workers and increased the unemployment rate [42, 43]. The mental health status of individuals with financial insecurity may have been especially worse than that of the general population during the pandemic [44]. Thus, in the present study, the participants with poor mental health owing to financial insecurity, but not with severe mental distress, may have been hesitant to order in, which is generally more expensive than home-cooked meals in Japan. In addition, some participants who continued to refrain from going out may have experienced mild distress owing to fewer opportunities to enjoy eating out [45, 46].

Poor mental health showed a significant association with decreased outings, seeing friends, and hospital visits in the present study. A previous study among Japanese healthcare workers revealed that decreased communication with friends during the pandemic was associated with poor mental health, indicating that loneliness could be a factor that explains the association [47]. In addition, because physical separation as well as decreased communication may have caused social isolation and poor mental health among individuals [48, 49], the results presented above would have been observed in the present study.

Age-stratified analyses revealed that the ORs of the older participants in the 5≤K6<9 and K6≥9 groups were higher than those of the younger ones for almost all undesirable and desirable lifestyle changes in the present study. A community-based study of Japanese adults reported that participants aged >60 years who spent more time at home, spent less time shopping in stores, or had fewer opportunities to exercise outdoors during the pandemic were significantly more likely to experience psychological distress compared with those aged <60 years [50]. These results are consistent with those of the present study.

The lifestyle changes among those with poor mental health status observed in this study may not be specific to the COVID-19 pandemic. For example, the previous studies before the pandemic also reported the associations between poor mental health and lifestyle changes such as increased alcohol consumption [5] and overeating [6]. However, this study is unique in the following way: during the COVID-19 pandemic in Japan, people were requested to refrain from non-essential going out and activities, and this restricted peoples’ daily lives. The results of the present study suggest that mental assessment may be one of the good ways to find people who tend to deteriorate in their lifestyles during the above-mentioned special situation, and also suggest the significance of this cross-sectional study. Furthermore, several previous studies have reported that physical activity [51, 52] and online psychological interventions [53] improved mental health during the COVID-19 pandemic. Accordingly, these results, together with the results of the present study, suggest that working on improving mental health helps prevent the deterioration of lifestyle among individuals with poor mental health in pandemics. However, since the present study was cross-sectional in design, whether improving one’s mental status during a pandemic results in improvement in his/her lifestyle remains to be determined with future studies.

Several previous studies have investigated the association between mental health status and lifestyle changes during the pandemic; however, most of these studies were web-based studies conducted by specific websites or social network services. The present study is valuable in that it is one of the few reports to investigate the association in the nationwide general population. In addition, the present study revealed dose-response relationships between mental health status and lifestyle changes even in the Japanese population, wherein the incidence of COVID-19 was lower, and behavioral restrictions without legal penalties were less severe compared with those in the Western population. Among the lifestyles, the ORs for increased alcohol drinking and decreased physical activity were especially high. These results indicate that individuals with a poor mental health, as assessed using the K6 score, could be at an especially high risk of increased alcohol drinking and decreased physical activity during pandemics. Implementing measures for the prevention of the spread of infection should be a priority during pandemics. However, it would be important for health care providers to assess mental status of individuals using questionnaires, such as K6, and to evaluate their lifestyles, especially alcohol drinking and physical activity, in those with poor mental health to continue and maintain prevention of NCDs during future pandemics.

The present study has several limitations. Firstly, the directionality of the associations between mental status and lifestyle changes was difficult to determine as this was a cross-sectional study. Secondly, information on the socioeconomic factors during the pandemic that may confound the association was lacking [44, 54]. Thirdly, lifestyle changes were assessed using subjective questionnaires, and participants with poor mental health may have evaluated their lifestyle changes more negatively than those without poor mental health [55]. Fourthly, the frequencies or degrees of lifestyle changes were not investigated in this study; therefore, we could not give more details on lifestyle changes in the results. Lastly, the associations observed in the present study were limited to the COVID-19 pandemic.

## Conclusions

Poor mental health was associated with undesirable lifestyle changes, especially increased alcohol drinking and decreased physical activity, in the general population in Japan during the COVID-19 pandemic. We suggest that health care providers need to pay attention to the mental status of individuals and to assess the lifestyles, especially alcohol drinking and physical activity, in those with poor mental health to continue and maintain the prevention of NCDs during future pandemics.

## Abbreviations

COVID-19: coronavirus disease 2019
NCDs: non-communicable diseases
NIPPON DATA2010: the National Integrated Project for Prospective Observation of Non-communicable Disease and its Trends in the Aged 2010
NHNS2010: the National Health and Nutrition Survey of Japan
K6: Kessler 6
MHLW: Ministry of Health, Labour and Welfare
OR: odds ratio
CI: confidence interval
SD: standard deviation
UPF: ultra-processed food

## Supplementary information

**Additional file 1:** **Supplementary Table 1** Sex-specific odds ratios of the K6 groups for undesirable lifestyle changes. **Supplementary Table 2** Odds ratios of the K6 groups for undesirable lifestyle changes by age group. **Supplementary Table 3** Sex-specific odds ratios of the K6 groups for desirable lifestyle changes. **Supplementary Table 4** Odds ratios of the K6 groups for desirable lifestyle changes by age group.

## Declarations

### Availability of data and materials

Data were provided from Ministry of Health, Labour and Welfare Japan and the authors do not have the right to share them.
